# Photocatalytic hydrogen production toward carbon neutrality: tracking charge separation

**DOI:** 10.1093/nsr/nwad139

**Published:** 2023-05-15

**Authors:** Bao-Lian Su

**Affiliations:** Laboratory of Inorganic Materials Chemistry, University of Namur, Belgium; State Key Laboratory of Advanced Technology for Materials Synthesis and Processing, Wuhan University of Technology, China

Under the vision of ‘carbon peaking and carbon neutrality’, the global energy system is facing a deep restructuring. As a clean energy with the potential to achieve zero carbon emissions, hydrogen energy will undoubtedly play an important role. Solar-energy-driven photocatalytic reactions can decompose water to produce hydrogen and reduce carbon dioxide to produce solar fuels. This major scientific challenge is particularly important for ‘carbon neutrality’ and has garnered significant global attention across the world.

Since the beginning of this century, research on photocatalytic hydrogen production has shown an accelerated development and gained significant progress. One representative advancement is the successful construction of the SrTiO_3_ and BiVO_4_ Z-scheme by Professor K. Domen from the University of Tokyo, Japan, which has increased the solar energy photocatalytic hydrogen production efficiency to >1% [1]. Moreover, based on the high quantum efficiency of the SrTiO_3_ powder catalyst, a demonstration device on a 100-m^2^ scale with an efficiency of 0.76% under natural light has been built, which operates safely and stably [2]. However, the solar hydrogen production efficiency still remains at a very low level far from the need for the industrialization.

The understanding of photoinduced charge transfer in photocatalysts and their transport from the interior to the surface reaction sites during photocatalytic hydrogen production is essential for the design and the synthesis of efficient and industrially scalable photocatalysts without using any sacrificial agents toward the industrialization. The mechanism of photoinduced charge separation thus attracts much research. One recent advance was made by researchers from the Dalian Institute of Chemical Physics, Chinese Academy of Sciences. They developed the surface photovoltage microscopic method with high spatial resolution and quantitatively studied the charge distribution on the photocatalytic particle at the nm/μm scale [3–5]. These studies provided valuable insight into the driving force for charge separation in photocatalyst particles.

However, the photocatalytic hydrogen production process spans time and space scales from femtoseconds to seconds and from atoms to micrometers, respectively, so uncovering the microscopic mechanism of this entire process is extremely challenging. The same group integrated multiple techniques that can be connected in time and space scales to detect the photoinduced charge transfer in photocatalytic nanoparticles [6–11]. First, home-built surface photovoltage microscopy (SPVM) clearly revealed that the construction of defects with spatial selectivity enables efficient spatial separation of photogenerated electrons and holes on different facets of the photocatalyst particles (Fig. 1a and b). Furthermore, they used time-resolved photoemission electron microscopy (tr-PEEM) to map the charge transfer on ultrafast timescales (Fig. 1c) and found that photoinduced electrons can selectively transfer from <111> to <010> crystal facet regions on a sub-picosecond timescale—an unexpected ultrafast timescale (Fig. 1d). They confirmed that the ultrafast charge transfer in this process originates from a new quasi-ballistic transport mechanism (Fig. 1e). Finally, they conducted transient photovoltage analysis (Fig. 1f) and found that as the timescale developed from nanoseconds to microseconds, holes gradually appeared on the defect-containing <111> facets (Fig. 1g). For the first time, the researchers combined three different methods—time-resolved photoemission microscopy, transient surface photovoltage spectroscopy and SPVM—in a relay race-like fashion to track the entire mechanism of electron and hole transfer to surface reaction centers in a single photocatalyst particle.

**Figure 1.**
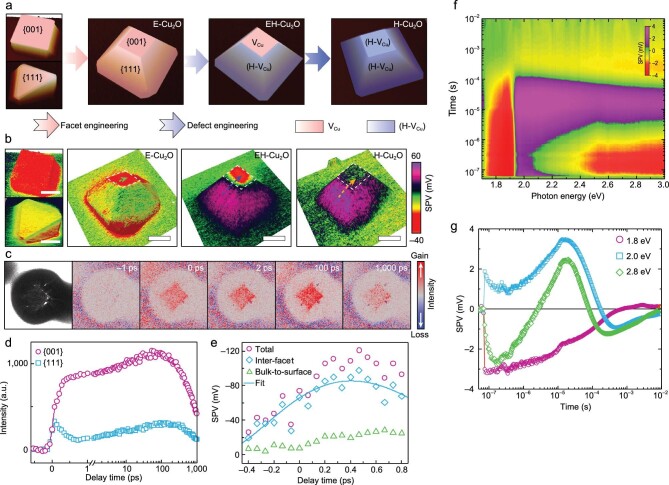
(a) Illustration of the anisotropic engineering of facets and defects of Cu_2_O photocatalyst particles [11]. (b) SPVM images of Cu_2_O photocatalyst particles with different facet and defect structures [11]. Scale bars: 2 μm. (c) tr-PEEM images of a Cu_2_O photocatalyst particle with anisotropic defects [11]. (d) Photoelectron intensity plotted as a function of delay time for Cu_2_O {001} and {111} facets [11]. (e) Decoupled ultrafast SPV signals induced by the inter-facet electron transfer with simulations via the quasi-ballistic model [11]. (f) Transient SPV spectra of Cu_2_O photocatalyst particles with anisotropic defects [11]. (g) SPV transients extracted from different photon energies in (f) [11]. Adapted with permission from Ref. [11]. Copyright 2022 Springer Nature.

Through the full spatio-temporal detection of photogenerated charge transfer in cuprous oxide photocatalyst nano single particles, a variety of different techniques are integrated: including tr-PEEM, transient photovoltage and SPVM, which reveals the complex microscopic process of multiple charge-transfer mechanisms and ‘photographs’ a full spatio-temporal map of the photogenerated charge-transfer evolution of nanoparticle photocatalysts. The essential correlation between these charge-separation mechanisms and photocatalytic water-splitting efficiency is clarified, which provides a new understanding and research strategy for breaking through the ‘bottleneck’ of charge separation in photocatalytic water-splitting catalysts.

These studies constitute a significant breakthrough in the fundamental understanding of photocatalysis, displaying high levels of originality. The approach used to track charge transfer and distribution with high spatial and temporal resolution at the single-particle level provides a novel research method for photocatalytic processes. Such a method will greatly advance the understanding of the complex mechanisms involved in photocatalysis, enabling the diagnosis of bottleneck problems. These new pieces of knowledge will guide the development of innovate strategies for synthesizing efficient and industrially scalable photocatalysts toward the industrialization of photocatalytic hydrogen production, which makes the hydrogen-based energy system a reality, being a great contribution to achieving ‘carbon neutrality’.

## References

[1] Wang Q , Hisatomi T, Jia QX et al. Nat Mater 2016; 15: 611–5. 10.1038/nmat4589 26950596

[2] Nishiyama H , Yamada T, Nakabayashi M et al. Nature 2021; 598: 304–7. 10.1038/s41586-021-03907-3 34433207

[3] Chen RT , Fan FT, Dittrich T et al. Chem Soc Rev 2018; 47: 8238–62. 10.1039/C8CS00320C 30059114

[4] Gao YY , Cheng F, Fang WN et al. Natl Sci Rev 2021; 8: nwaa151. 10.1093/nsr/nwaa151 34691655 PMC8288172

[5] Gao YY , Nie W, Zhu QH et al. Angew Chem Int Ed 2020; 59: 18218–23. 10.1002/anie.202007706 32671941

[6] Zhu J , Pang S, Dittrich T et al. Nano Lett 2017; 17: 6735–41. 10.1021/acs.nanolett.7b02799 28967261

[7] Chen RT , Fan FT, Li C. Angew Chem Intl Ed 2022; 61: e202117567. 10.1002/anie.202117567 35100475

[8] Liu Y , Ye S, Xie HC et al. Adv Mater 2020; 32: 1906513. 10.1002/adma.201906513 31943380

[9] Liu Y , Zhang MJ, Wang Z et al. Nat Commun 2022; 13: 4245. 10.1038/s41467-022-32002-y 35869136 PMC9307613

[10] Chen RT , Rang S, An HY et al. Nat Energy 2018; 3: 655–63. 10.1038/s41560-018-0194-0

[11] Chen RT , Ren Z, Liang Y et al. Nature 2022; 610: 296–301. 10.1038/s41586-022-05183-1 36224420

